# The Hidden Link of Exosomes to Head and Neck Cancer

**DOI:** 10.3390/cancers13225802

**Published:** 2021-11-19

**Authors:** Yong Teng, Lixia Gao, Reid Loveless, Juan P. Rodrigo, Primož Strojan, Stefan M. Willems, Cherie-Ann Nathan, Antti A. Mäkitie, Nabil F. Saba, Alfio Ferlito

**Affiliations:** 1 Department of Hematology and Medical Oncology, Winship Cancer Institute, Emory University School of Medicine, Atlanta, GA 30322, USA; nfsaba@emory.edu; 2 National & Local Joint Engineering Research Center of Targeted and Innovative Therapeutics, College of Pharmacy, Chongqing University of Arts and Sciences, Chongqing 402160, China; lx126001@126.com; 3 Department of Oral Biology and Diagnostic Sciences, Augusta University, Augusta, GA 30912, USA; reloveless@augusta.edu; 4 Department of Otolaryngology, Hospital Universitario Central de Asturias (HUCA), Instituto Universitario de Oncología del Principado de Asturias (IUOPA), University of Oviedo, 33011 Oviedo, Spain; jprodrigo@uniovi.es; 5 Spanish Biomedical Research Network Centre in Oncology, CIBERONC, 28029 Madrid, Spain; 6 Department of Radiation Oncology, Institute of Oncology Ljubljana and Faculty of Medicine, University of Ljubljana, 1000 Ljubljana, Slovenia; pstrojan@onko-i.si; 7 Department of Pathology and Medical Biology, University of Groningen, 9727 Groningen, The Netherlands; s.m.willems@umcg.nl; 8 Department of Otolaryngology-Head and Neck Surgery, Louisiana State University-Health Shreveport, Shreveport, LA 71103, USA; cherieann.nathan@lsuhs.edu; 9 Department of Otorhinolaryngology-Head and Neck Surgery, University of Helsinki and Helsinki University Hospital, FI-00029 Helsinki, Finland; Antti.Makitie@hus.fi; 10 Coordinator of the International Head and Neck Scientific Group, 35125 Padua, Italy; profalfioferlito@gmail.com

**Keywords:** exosomes, head and neck squamous cell carcinoma, the tumor microenvironment, biomarker, therapeutic targets

## Abstract

**Simple Summary:**

Head and neck squamous cell carcinomas (HNSCC) are a challenging group of malignancies that require a multidisciplinary management approach and where advances in targeted therapeutics remain relatively meager. Despite these challenges, recent advances in immunotherapeutic approaches have positively affected the clinical outcome for patients with advanced disease. Novel approaches for systemic therapy continue, however, to be an urgent need for this disease. This review highlights the possible role of exosomes in sustaining malignant cells in HNSCC and the prospects of future therapeutic interventions in these cellular components based on current evidence.

**Abstract:**

Head and neck squamous cell carcinoma (HNSCC) represents an aggressive and heterogenous group of cancers whose pathologies remain largely unresolved. Despite recent advances in HNSCC therapeutic strategies, the overall survival of HNSCC patients remains poor and continues to prompt efforts to develop more effective therapies. Exosomes are a subtype of extracellular vesicles secreted by a variety of cells that have begun to spark significant interest in their roles in cancer. As membranous vesicles, spanning from 30–150 nm in diameter, exosomes mediate the transport of various molecules, such as proteins, nucleic acids, and lipids, intercellularly throughout the body. In doing so, exosomes not only act to deliver materials to cancer cells but also as signals that can confer their progression. Accumulating evidence shows the direct correlation between exosomes and the aggressiveness of HNSCC. However, more research is warranted in this field to further our understanding. In this review, we attempt to highlight the tumor-supporting roles and therapeutic potential of exosomes in HNSCC. We introduce first the biogenesis and component features of exosomes, followed by their involvement in HNSCC proliferation and metastasis. We then move on to discuss HNSCC-derived exosomes’ influence on the tumor microenvironment and their function in tumor drug resistance. Finally, we explore the promising potential of exosomes as HNSCC biomarkers and therapeutic targets and drug carriers for HNSCC treatments.

## 1. Introduction

Head and neck cancer is the sixth most common cancer worldwide [1,2,3]. More than 90% of all head and neck cancers are classified as head and neck squamous cell carcinoma (HNSCC), with a high frequency of tumor recurrence/metastasis and low patient survival. In 2018 alone, there were a reported 890,000 new cases of HNSCC and 450,000 associated deaths globally [1,2,3]. HNSCC comprises a heterogeneous group of cancers derived from the mucosal epithelium of the oral cavity, pharynx, and larynx and is typically linked to tobacco consumption, alcohol abuse, and human papillomavirus (HPV) infection. Despite advances in HNSCC surgical treatment, chemoradiotherapy, and immunotherapy, more than two-thirds of HNSCC patients still have no way to effectively control their clinical progression, facing a 5-year survival rate of about 60% [4]. Therefore, exploring new therapeutic targets for the treatment of HNSCC is of great significance. Here, we discuss recent studies on the biological functions of exosomes in HNSCC, including their essential roles and value as potential targets in HNSCC therapy.

Exosomes are small membranous vesicles that act as intercellular messengers through the cargo they carry. These discoid vesicles are 30–150 nm in diameter and secreted by living cells from early endosomes containing a typical lipid bilayer structure [5]. Their distribution is widespread, and they are found in various bodily fluids, such as serum, plasma, saliva, urine, and amniotic fluid [6]. Through their transportation of lipids, proteins, and nucleic acids, exosomes play an indispensable role in transferring material and information between cells and are consequently expected to serve as early diagnostic markers for various diseases [7,8].

## 2. Exosomes

### 2.1. Biogenesis of Exosomes

**Figure 1:**
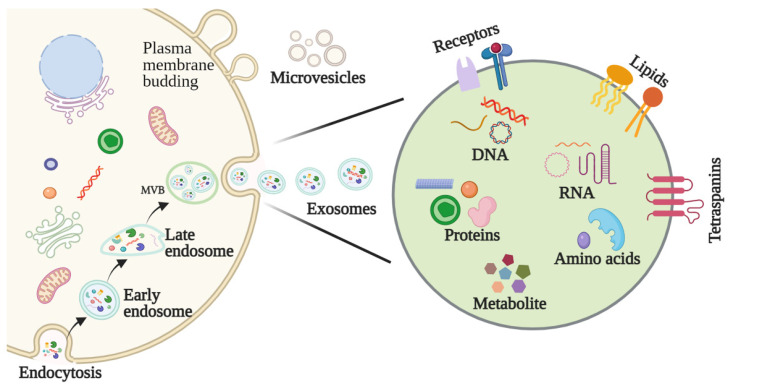
Schematic presentation of the process by which exosomes are produced and released from the cell to the TME.

The biological origins of exosomes begin with early endosomes, which form through plasma membrane invagination. During this process, extracellular components and cell membrane proteins are encapsulated, and inward budding of the early endosome’s membrane leads to the formation of an exosomal vesicle. Early endosomes not only exchange materials with other organelles but fuse with different endosomes to form late endosomes. Late endosomes with intraluminal vesicles are known as multivesicular bodies (MVBs), and these are responsible for fusing with cell membranes to release the exosomes into the extracellular environment (Figure 1) [8,9].

### 2.2. Features and Components of Exosomes

Exosomes, microvesicles, microparticles, and apoptotic bodies are subtypes of extracellular vesicles (EVs) [10]. While these vesicles share similar characteristics, they differ in their morphologies, biological properties, biogenesis, and functional roles. Exosomes are structurally similar to their cells of origin, possess a lipid bilayer structure, and typically consist of lipids, proteins, nucleic acids, and metabolites [11]. Exosomes also notably possess specific surface proteins that can be used to discern whether they are tumor derived (Figure 1) [12].

### 2.3. Methods for Exosome Isolation and Characterization

The isolation of pure exosomes is the first critical step in studying their mechanisms of action and their application in biomedical sciences. Various techniques have been adopted to facilitate the isolation of exosomes, including ultracentrifugation, size-based filtration, size-exclusion chromatography, polymer precipitation, and microfluidics-based isolation. Because exosome membranes are known to contain large quantities of proteins, immunoaffinity methods are also suitable for isolating exosomes [13].

Characterization of the physicochemical properties of exosomes, including their size, shape, surface charge, density, and porosity, is also important to their functional determinations. Many techniques have been routinely used to characterize exosomes, including dynamic light scattering (DLS), tunable resistive pulse sensing (TRPS), flow cytometry, electron microscopy, nanoparticle tracking analysis (NTA), and atomic force microscopy (AFM) [13]. Due to the inherent heterogeneity of exosomes and the advantages and limitations of each technique, more sophisticated techniques are necessary for the isolation and characterization of exosomes.

Owing to the stability of exosomes in the circulation and body fluids, they have become attractive as potential biomarkers of different diseases. Saliva is a body fluid and a mixture of the secretions from the major salivary glands that may represent a valuable source of diagnostic and therapeutic markers in HNSCC patients. Ogawa et al. were among the first to identify salivary exosomes in 2008 [14]. This group also revealed the presence of two different types of exosomes with different mean diameters (I: 83.5 nm, II: 40.5 nm) and protein constituents [15]. Exosomes isolated from individual salivary glands are derived from cells within that specific gland (including parotid, submandibular, and sublingual), which may reflect the physiologic state of the gland at both protein and regulatory levels. To assess the microRNA content of exosomes, exosomal pellets are treated with RNaseA, followed by miRNA isolation from exosomal lysates with a kit that also preserves mRNA. Typically, saliva samples ranging from 200 uL up to 5 mL volume yield an adequate amount of exosomal RNA for quantitative PCR [16]. To establish a standardized collection protocol for the isolation of salivary exosomes, Ohshiro et al. compared three different processing techniques that are used to prepare saliva specimens prior to liquid chromatography–tandem mass spectrometry (LC-MS/MS) analysis [17]. They concluded that initial saliva processing affected protein analysis and in-gel digestion followed by LC-MS/MS detection yielded the highest number of proteins. Using this approach, they successfully identified that α-1-B-glycoprotein and complement factor B proteins were only present in saliva from HNSCC patients when compared with healthy individuals.

## 3. The Function of Exosomes in HNSCC

The study of exosomes in cancer has rapidly grown in recent years compared to studies in other diseases [18]. Exosomes are reported to influence tumorigenesis, proliferation, migration, and drug resistance, and their role in tumor progression is closely tied to tumor type, genetics, and cancer stage [19,20]. As cargo carriers capable of transporting molecular components, such as cell receptors, transcription factors, and enzymes, exosomes facilitate a broad range of tumor-supporting functions across different cells [21,22]. For instance, studies have shown that tumor-specific markers of tumor-derived exosomes (TDEs) can promote cancer progression by stimulating angiogenesis [23,24]. As a redox sensor, oxidized ATM serine/threonine kinase (ATM) has been linked to the maintenance of cellular redox homeostasis. Most recently, we and others identified that oxidized ATM has the potential to induce autophagosome accumulation and exosome release from hypoxic breast cancer-associated fibroblasts (CAFs) through phosphorylating BCL2-interacting protein 3 (BNIP3) [25]. Moreover, we found that enriched autophagy-associated GPR64 in hypoxic CAFs-derived exosomes upregulates MMP9 and IL-8 in recipient breast cancer cells via non-canonical NF-κB signaling, which is required for breast cancer cells to gain invasive abilities [25]. Interestingly, PD-L1 was also found on the exosome’s surface, and exosomes expressing PD-L1 can inhibit antitumor immune responses. Shimada et al. measured serum exosomal PD-L1 and tumor PD-L1 expression to understand anti-PD-1 response and clinicopathological outcomes in non-small cell lung cancer (NSCLC). In this study, they found that exosomal PD-L1 levels were strongly correlated with tumor PD-L1 levels and worse relapse-free survival in NSCLC patients, suggesting exosomal PD-L1 represents a reliable biomarker to predict anti-PD-1 response and clinical outcomes in patients with NSCLC [26]. Lysyl oxidase-like 4 (LOXL4) LOXL4 is commonly upregulated in hepatocellular carcinoma (HCC) tissues. A recent study shows that HCC-derived exosomes transferred LOXL4 between HCC cells and to human umbilical vein endothelial cells (HUVECs) and promoted cancer cell invasion, metastasis, and angiogenesis [27].

Besides proteins, many studies have demonstrated that mRNAs, miRNAs, and other noncoding RNAs are contained in exosomes. For example, Chiba and his colleagues found that exosomes derived from colorectal cancer cells contained mRNAs, miRNAs, and natural antisense RNAs were delivered into recipient cells to support tumor development [28]. The diverse molecular cargos are selectively absorbed when exosomes circulate, leading to a modification in the target gene expression, signal, and overall biological function of recipient cells under physiological or pathological conditions. It has been reported that more than 90% of extracellular miRNA are indeed outside EVs and associated with proteins of the Argonaute (AGO2) family both in blood plasma/serum and cell culture media [29].

Exosomes from HNSCC are similar to those from other tumors in that they contain a variety of bioactive substances that enable them to alter the function of recipient cells and the broader tumor microenvironment (TME) [30]. The functions associated with HNSCC-derived exosomes reported to date are highlighted in Table 1 and discussed in greater detail in subsequent sections. Researchers are continuing to explore the precise roles of exosomes in HNSCC.

### 3.1. Exosomes Affect HNSCC Growth

Angiogenesis is critical to tumor cell proliferation, and, in one study, TGF-β-containing HNSCC-derived exosomes were found to promote angiogenesis in vitro and in vivo [31]. Additionally, Wang et al. reported that CAF-derived exosomes containing miR-3188 can influence the proliferation of HNSCC cells in vitro and in vivo, which suggests a potential therapeutic value for exosome-delivered miR-3188 in inhibiting HNSCC growth [32]. While investigating the diagnostic potential of salivary exosomal miRNAs as screening biomarkers for oral squamous cell carcinoma (OSCC), researchers also found that exogenous exosome miR-24-3p increases recipient malignant cell proliferation through targeting of PER1 protein [33].

### 3.2. Exosomes Are Involved in HNSCC Invasion and Metastasis

Epithelial to mesenchymal transformation (EMT) is an important feature of tumor progression and promotes the invasion and metastasis of tumor cells into the stroma. Exosome-mediated intercellular communication contributes significantly to this process and investigating it has led to breakthroughs in the field of cancer metastasis [39]. One study has shown that PO-243 exosomes from the plasma of HNSCC patients undergoing photodynamic therapy can alter the mesenchymal characteristics of tumor cells, directing them towards epithelial phenotypes [34]. Another study evaluated immunocaptured CD44v3+ tumor cell-derived exosomes from the plasma of 44 HNSCC patients and 7 healthy donors and found higher immunosuppressive protein levels in CD44v3+ exosomes compared with CD44v3(-) exosomes. The relative fluorescence intensity of these markers was associated with higher disease stages and lymph node metastasis [35].

### 3.3. Exosomes Regulate the HNSCC Microenvironment

#### 3.3.1. Exosomal Modulation of the Pre-Metastatic Niche (PME)

The TME is an arena of dynamic and diverse components, including tumor cells, stromal cells (e.g., cancer-associated fibroblasts (CAFs), immune cells (e.g., tumor-associated macrophages), vasculature, the extracellular matrix, and secretory products, among others [40]. Tumor development is tied closely to changes in the tumor stroma. Through the secretion of various factors and cytokines, tumors strategically tailor their microenvironment to support their growth, proliferation, and dissemination [41].

How a tumor induces PMN formation in a specific organ remains to be determined. The suppressive nature of immune cells in the TME is critical to the regulation of anti-tumor immune responses. One of the possible mechanisms is that TDEs mediate tumor PMN remodeling to establish a supportive and receptive niche to promote tumor cell colonization and metastasis. Maybruck et al. have found, however, that head and neck cancer cells can induce a suppressive phenotype in human CD8^+^ T cells through the release of TDEs [42]. Specifically, the group revealed through mass spectrometry that the immunoregulatory protein galectin-1 was present in these exosomes and played a key role in inducing this suppressive phenotype. The purification of exosomal RNA and subsequent CD8^+^ T cell suppression analysis also implicated RNAs in T cell dysfunction. This study suggests that tumor immunosuppressive exosomes could be a potential therapeutic target to preserve T cell function in anti-tumor immune responses [42]. Ludwig et al. isolated exosomes by size-exclusion chromatography from the plasma of 38 HNSCC patients and 14 healthy donors and measured exosome-mediated effects on the functions of normal human lymphocyte subsets and natural killer (NK) cells [43]. They found that the presence, quantity, and molecular content of isolated, plasma-derived exosomes discriminated HNSCC patients with active disease (AD) from those with no evident disease (NED) after oncological therapies. Compared with exosomes of patients with NED, those of patients with AD were significantly more effective in inducing apoptosis of CD8^+^ T cells, suppressing CD4^+^ T cell proliferation, and up-regulating of regulatory T cell (Treg) suppressor functions. Additionally, exosomes of AD patients also downregulated NKG2D expression levels in NK cells, suggesting exosome-induced immune suppression is associated with disease activity in HNSCC [43].

#### 3.3.2. Exosomal Modulation of Tumor Hypoxia

Hypoxia is one of the common characteristics of solid tumors, and it is currently believed that a modulatory relationship exists between hypoxia and TDEs [36]. Studies have shown, for instance, that hypoxia can influence the role of γδ T cells in tumors by altering the content of TDEs [44]. Repression of γδ T cell expansion and cytotoxicity under hypoxia by TDEs was found to be HSP70-dependent and a result of enhanced myeloid-derived suppressor cell activity through a miR-21/PTEN/PD-L1 regulatory axis. The combination of miR-21 and PD-L1 targeted therapy was also seen to have a positive therapeutic effect in immunocompetent mice with OSCC [44]. The PD-1/PD-L1 pathway is well known for its roles in various immunosuppressive mechanisms of HNSCC. Related studies have shown that the levels of PD-L1-containing exosomes in the plasma of HNSCC patients, though not serum, correlated with the patients’ disease activity, stage, and lymph node status [45]. Additionally, exosomes secreted by OSCC cells have been reported to activate macrophages through p38, Akt, and SAPK/JNK signaling shortly after their uptake [46]. Specifically, the exosomal-derived adhesive glycoprotein THBS1 was found to participate in the polarization of macrophages to an M1-like phenotype. Treating OSCC cells with culture media from exosome-activated macrophages also significantly promoted OSCC cell motility [46]. Still, further study is warranted to delineate the ways exosomes contribute to macrophages’ functional phenotypes and understand their associated microenvironmental consequences.

#### 3.3.3. Exosomal Modulation of Immune Escape and Suppression

Modulation of the immune system is one of the hallmarks of cancer. Increasing evidence has highlighted how exosomes represent a mechanism underpinning cancer cell-triggered immune escape by inducing phenotypic changes in different immune cell populations. Paula Silva et al. evaluated the potential effects of EVs originating from HNSCC cells on immune system response [47]. In this study, monocyte-derived dendritic cells (mono-DCs) were treated with EVs derived from oropharyngeal squamous cell carcinoma (OPHSCC) and OSCC cell lines [47]. Impairment of mono-DC maturation and migration was observed following EV internalization. Gene expression profiling in mDCs treated with EVs further reveals disrupted immune responses possibly targeted by miRNAs (e.g., miR-17-5p and miR-21) within EVs [47]. To test whether tumor- or T cell-derived exosomes in HNSCC patients’ plasma are immunosuppressive and impact disease activity, Theodoraki’s group separated CD3(–) from CD3(+) exosomes by immunocapture using anti-CD3 antibodies [48]. They concluded that the CD3(–) exosome fraction, which is enriched in TDEs, emerges as a source of information about the role of the TDE molecular content in the progression of HNSCC.

### 3.4. Exosomes Promote Drug Resistance in HNSCC

Despite advances in our understanding, tumor resistance to traditional chemotherapy drugs remains one the greatest contributors to poor therapeutic responses and must be addressed to improve patient outcomes [49]. One way by which exosomes contribute to tumor drug resistance is through their sequestering and efflux of cytotoxic drugs, effectively shielding cells from the drug’s effect and preventing its intracellular accumulation [50]. Exosomes can also contribute to external therapeutic resistance through their mediation of intercellular communication and delivery of mRNAs, miRNAs, DNAs, and/or proteins, for example [51]. Qin et al. have found that CAFs are inherently resistant to cisplatin and that exosomes act to transfer functional miR-196a from CAFs to HNSCC cells. In doing so, HNSCC cell proliferation and resistance to apoptosis are conferred through the targeting of CDKN1B and ING5, two suggested tumor suppressors [37]. Hence, the group put forward that miR-196a may represent a promising biomarker and/or potential therapeutic target for cisplatin-resistant cancers [37].

Additionally, Khoo et al. observed an increased EV production in de novo (H314) and adaptive (H103/cisD2) cisplatin-resistant OSCC cell lines compared to an H103-sensitive cell line [52]. ATP1B3, a metal ion transporter, was also found to be downregulated in the drug-resistant cells, indicating altered drug delivery. Further study revealed that while the drug-resistant cells accumulated less cisplatin than the sensitive cells, higher levels were found in their EVs than those of sensitive cells. Using a proton pump inhibitor to prevent the cellular efflux of EVs, the group further showed they could increase the drug sensitivity of the cisplatin-resistant H314 cells. Therefore, working to control the release of HNSCC cell-derived exosomes may be an effective approach to combating HNSCC drug resistance [52].

## 4. Role of Exosomes in the Diagnosis and Treatment of HNSCC

### 4.1. Exosomes as a Potential Biomarker in HNSCC

**Figure 2:**
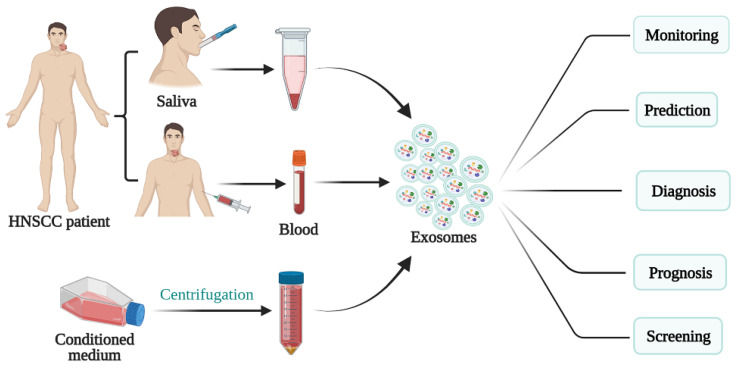
Schematic presentation of the use of exosomes for HNSCC diagnosis, prognosis, and treatment.

Early diagnosis and treatment are critical determinants in a cancer patient’s prognosis. The application of biomarkers in HNSCC detection, as well as factors such as staging, treatment efficacy, and prognosis, have consequently garnered attention in recent years [53]. Biomarkers represent a diverse range of molecules, and abnormalities in their levels or makeup can be detected in bodily fluids, like urine, saliva, and blood, as well as tumors themselves. In particular, exosomal biomarkers may be roughly divided into nucleic acids, proteins, lipids, and metabolites (Figure 2). Interestingly, it has been demonstrated through infrared signature absorption and computer-aided analysis that subtle differences between the conformation of proteins, lipids, and nucleic acids in oral cancer salivary exosomes and healthy salivary exosomes can be detected and used to differentiate the two [54].

Exosomal miRNAs are commonly found in HNSCC liquid biopsies, and many groups have reported certain miRNAs (e.g., miR-142, miR-186, miR-31) circulating at high levels to constitute potential prognostic biomarkers [55]. Studies have also confirmed that tumor cell-derived exosomes can secrete heat shock proteins (HSP), which have been found in significantly higher levels in HNSCC patients, and could be used as biomarkers for cancer metastasis, for instance [56,57]. As previously mentioned, CD44v3+ tumor cell-derived exosomes in HNSCC patients were found to carry higher levels of immunosuppressive proteins than CD44v3(-) exosomes [35]. Because CD44v3 overexpression is associated with poorer patient outcomes, the glycoprotein may prove to be a useful biomarker in HNSCC [35]. On the other hand, decreased levels of proteins such as ANXA1, which has been shown to play a tumor-suppressing role in HNSCC through its regulation of EGFR activity and exosomal phospho-EGFR release, may also represent important prognostic biomarkers [58]. Moreover, it is likely that exosomal miRNAs linked to EGFR, which is overexpressed in 90% of HNSCC, or those associated with other factors tied to poor patient prognosis, such as HPV, hold significant value in their potential to monitor HNSCC progression and guide its treatment [55,59,60].

Oral lichen planus (OLP) is a chronic inflammatory disease of the oral mucosal lining. Byun et al. revealed that salivary exosomal miRNAs, such as miR-4484, miR-1246, and miR-1290, are candidates for diagnosing and elucidating the pathogenesis of OLP, as their expression profile in patients with OLP is distinct from that in healthy controls [61]. Langevin and his colleagues employed microRNA-sequencing (miRNA-seq) to catalog differentially secreted exosomal miRNA from HNSCC cells relative to non-pathologic oral epithelial cells [62]. Interestingly, 32 exosomal miRNAs across four HNSCC cell lines that originated from different sites in the upper aerodigestive tract were dysregulated compared with their normal counterparts. Those miRNAs included miR-486-5p and miR-10b-5p, which were further validated by droplet digital PCR in an independent set of saliva samples from an additional 11 HNSCC patients and 9 cancer-free controls. Thus, this study yields a novel set of candidate exosomal miRNAs that may have utility as non-invasive salivary biomarkers of HNSCC [62]. Another group identified two miRNAs (miR-302b-3p and miR-517b-3p) selectively enriched in EVs of OSCC patients [63]. These two salivary EV-associated miRNAs have potential clinical utility, as they could be applied as promising biomarkers for OSCC. Moreover, this study also suggests that salivary EVs isolated by a simple charge-based precipitation technique can be exploited as a non-invasive source of miRNAs for OSCC diagnosis [63].

In a pilot study, Theodoraki and his colleagues analyzed the potential of circulating exosomes as early detectors of treatment success in a cohort of locally advanced HNSCC patients undergoing conventional treatment. By comparison with patients’ plasma-derived exosomes from defined time-points before, during, and after therapy, they revealed that these collected exosomes can be considered as promising non-invasive biomarkers that are able to discriminate early in the course of treatment between patients who can be cured by surgery/(C)RT and those who will develop relapse [64].

### 4.2. Exosomes as Therapeutic Targets in HNSCC

Despite recent improvements in HNSCC treatment, the long-term survival rate of advanced HNSCC patients remains less than 50% [65]. Given their diverse roles in cancer support, from proliferation to metastasis, exosomes and the cargo they carry constitute an innumerable set of potential targetable points for influencing HNSCC progression [66,67]. Many studies have indicated that exosomes are involved in anticancer therapy resistance due to their critical role in cellular communication and the TME. We have established a theory that the upregulation of AKT signaling is the critical mechanism for radioresistance in HNSCC cells [68]. One mechanism behind this is that exosomes derived from irradiated HNSCC cells have the potential to upregulate the AKT pathway to promote migration and increase chemotaxis in recipient cancer cells [69]. Therefore, targeting the highly relevant exosomes may result in radiosensitivity. Strategies for inhibiting exosome-mediated tumor-promoting potential have focused on preventing exosome release and blocking the communication between tumor cells and recipient cells. Many pharmacological agents are being explored to investigate how best to block exosome excretion and trafficking. For example, GW4869 is a cell-permeable, symmetrical dihydroimidazolo-amide compound that acts as a non-competitive inhibitor of membrane neutral sphingomyelinase (nSMase). GW4869 has been relatively extensively studied and reported to inhibit exosomes, as it can interfere with lipid composition that is necessary for exosome shedding [70]. Indomethacin, a non-steroidal anti-inflammatory drug, has been shown to downregulate transcription of the ABCA3 transporter [71]. ABCA3 is an intracellular protein involved in lipid transport, and its inhibition impairs exosomes release. Thus, indomethacin may also be considered a potential agent for exosome inhibition. Due to the involvement of Ras during exosome release, Ras inhibitors are particularly associated with exosome trafficking. Among them, manumycin A, a farnesyltransferase inhibitor blocking the Ras pathway, has been studied as an inhibitor of exosome secretion [72]. However, secretion control, in vivo biological distribution, targeting specificity, and cell penetration in exosome-targeting cancer therapies remain challenging to HNSCC and other solid tumors. Interestingly, engineered exosomes have been applied to develop therapeutic anticancer vaccines for HPV-associated tumors [73]. Because a mutant of a human immunodeficiency virus 1 (HIV-1) Nef protein (Nefmut) has the potential to act as an exosome-anchoring element upon fusion with heterologous proteins, exosomes engineered in vitro were used to upload high amounts of HPV-E7 fused to Nefmut. As a result, the production of endogenously engineered exosomes led to an efficient anti-E7 cytotoxic T lymphocyte (CTL) immune response when injected in mice. Exosomes produced by tumor cells have been shown to reprogram functions of human immune cells. Ludwig et al. compared molecular cargos of exosomes isolated from supernatants of HPV (+) and HPV (−) HNSCC cell lines or from HNSCC patients’ plasma [74]. Their data suggest that TDEs retain molecular and viral features of their respective HPV (+) or HPV (−) parental cells, and these exosome-induced alterations affect immune reprogramming and might result in differential responses of HNSCC patients to oncological therapies.

### 4.3. Exosomes as Drug Carriers for HNSCC Treatment

Exosomes are recognized as one of the most promising anti-tumor therapeutic vectors due to their bilayer structure, biocompatibility, low immunogenicity, and ability to interact with target cells [75]. Given their limited bioavailability, however, synthetic exosome-mimics have been used in their place to mediate endogenous and exogenous delivery for cancer treatment [12]. In particular, Cohen et al. evaluated the parameters of exosomes derived from mesenchymal stem cells (MSC-exo) and A431 squamous cell carcinoma cells (A431-exo) as drug vectors and found MSC-exo to exhibit significantly greater tumor penetration and distribution in A431 tumor-bearing mice [38]. These results underscore that careful consideration should be given to an exosome’s type when applying it to drug delivery due to its unique properties and inherent targeting capabilities.

## 5. Prospects for Exosomes in Anticancer Therapy

Even with recent advances, HNSCC remains a major clinical challenge. Through their intercellular transmission of materials and signals in the local and distant TME, exosomes play a crucial role in promoting HNSCC proliferation, invasion, metastasis, drug resistance, and survival. Many studies have already pointed to exosomes as promising tumor biomarkers, and, through their employment in patients, improvements to HNSCC detection and treatment regimens may soon be a reality. Plant- and human tissue-derived exosomes have undergone clinical trials in lung cancer, melanoma, and colorectal [76,77,78,79]. Most recently, MSC exosomes containing siRNA targeting oncogenic KrasG12D mutations are being employed against pancreatic cancer in a clinical trial (NCT03608631). Still, exosomes have yet to be clinically applied in HNSCC therapies. This may be due in part to the high cost of purification and isolation of exosomes and a lack of understanding of the complex effects resulting from HNSCC-derived exosomes [80]. Therefore, it is imperative that scientists continue to improve purification and isolation technologies and delineate exosome’s specific mechanisms in HNSCC progression in order to harness them as tools to improve patient outcomes.

## 6. Conclusions

As a critical mediator between cells, exosomes play important roles in extracellular communication in normal and pathological processes. Particularly, exosomes secreted from tumor cells shuttle signaling molecules, including nucleic acids, proteins, and metabolites, from the primary tumor to distant organs to reprogram the recipient cells and drive tumor growth and metastasis. Cancer-derived specific exosomes hold great potential for the early diagnosis of cancers and represent a promising approach in cancer therapy. Moreover, the precise identification of exosomes and their cargos may be a breakthrough to capture the dynamic complexity of cancer, providing new insight into the development of therapeutic targets. Nevertheless, how to separate, expand and identify clinically relevant exosomes continues to be a challenge, especially regarding HNSCCs, as they are highly heterogeneous cancers. Another major challenge is to understand the mechanisms that regulate the heterogeneity of cancer exosomes. Despite these concerns, future efforts to develop exosome-based new therapeutics and diagnostics to improve the survival of cancer patients are warranted.

## Figures and Tables

**Table 1:** The function of exosomes in HNSCC.

Resource	Cell Line/Model/Tissue	Main Exosome Components	Setting	Associated Consequence (s)	Ref
Blood	4-NQO mice	TGF-β	in vitro: human, in vivo: mice	Promote angiogenesis and cancer progression	[31]
Plasma	BALB/C nude mice	miR-3188	in vitro: human, in vivo: mice	Promote cancer progression	[32]
Salivary	HSC6 and SCC25/OSCC patients	miR-24-3p	in vitro: human, in vivo: mice	Promote cancer progression	[33]
Plasma	HNSCC cell lines	po-243	in vitro: human, in vivo: mice	Enhance epithelial-mesenchymal transition	[34]
Plasma	nonmalignant or HNSCC cell lines	CD44v3+	in vitro: human, in vivo: mice	Promote disease stages and lymph node metastasis	[35]
Plasma	immunocompetent mice	miR-21	in vitro: human, in vivo: mice	Regulation of TME	[36]
Cell medium	cisplatin-resistant HN4-res and cisplatin-sensitive HNSCC cells (Cal27, SCC25 and HN4)	miR-196a	in vitro: human, in vivo: mice	Induce chemoresistance	[37]
Cell medium	mouse	mesenchymal stem cells exosomes and A431 squamous cell exosomes	in vivo: mice	delivery vehicles	[38]
